# Expression and significance of microRNA‐126 and VCAM‐1 in placental tissues of women with early‐onset preeclampsia

**DOI:** 10.1111/jog.14732

**Published:** 2021-03-10

**Authors:** Beibei Liu, Ling Liu, Shihong Cui, Yue Qi, Tiantian Wang

**Affiliations:** ^1^ Department of Obstetrics and Gynecology The Third Affiliated Hospital of Zhengzhou University Zhengzhou China

**Keywords:** microRNA, preeclampsia, placenta, VCAM‐1

## Abstract

**Objective:**

To investigate the expression of microRNA‐126 (miR‐126) and vascular endothelial cell adhesion molecule‐1 (VCAM‐1) in the placental tissues of women with early‐onset preeclampsia (EOPE) and their effects on trophoblast invasion.

**Materials and Methods:**

The placental tissues of 30 pregnant women with EOPE who delivered in the Third Affiliated Hospital of Zhengzhou University from November 2019 to May 2020 were selected as the preeclampsia (PE) group, and the placental tissues of 30 healthy pregnant women with normal prenatal examination were selected as the normal group. Immunohistochemistry was used to localize VCAM‐1 in placental tissues，the expression of miR‐126 and VCAM‐1 in placenta tissues of two groups and HTR‐8/SVneo cells transfected with miR‐126 were detected by real‐time polymerase chain reaction (RT‐PCR) and Western blot, and the correlation between them was analyzed. The invasion ability of cells transfected with miR‐126 was observed by Transwell invasion test.

**Results:**

Compared with the normal group, the expression of miR‐126 was higher and VCAM‐1 was lower in the placental tissues of the PE group, and the difference were statistically significant (*p* < 0.01). Moreover, VCAM‐1 was negatively correlated with the expression of miR‐126 (*r* = −0.391, *p* < 0.05). In vitro experiment, the expression level of VCAM‐1 in miR‐126 mimics transfection group was decreased, and the expression level of VCAM‐1 in miR‐126 inhibitor transfection group was increased; the invasion ability of HTR‐8/SVneo cells transfected with miR‐126 mimics was decreased, and the invasion ability of HTR‐8/SVneo cells transfected with miR‐126 inhibitor was enhanced.

**Conclusion:**

There was a negative correlation between the expression of miR‐126 and VCAM‐1 in EOPE.MiR‐126 and VCAM‐1 may participate in the occurrence and development of EOPE by affecting the invasion ability of trophoblast cells.

## Introduction

Preeclampsia (PE) is a new onset of idiopathic hypertension syndrome after 20 weeks of gestation, which greatly affects and damages the health and life safety of mothers and infants, and can lead to 10%–15% maternal death.^1^ In developing countries，its incidence rate is 4%–18%, and it is the second leading cause of stillbirth and premature infant death,^2^resulting in 50 000–60 000 deaths every year.^3^According to its onset, time can be divided into early onset PE (EOPE,<34 weeks) and late onset PE (LOPE， ≥34 weeks). Clinical studies have shown that early‐onset PE has a higher risk of maternal health and fetal life than later‐onset PE.^4^At present, it is generally believed that placental shallow implantation caused by insufficient invasion of trophoblast and incomplete placental vascular remodeling plays a very important role in the pathogenesis of EOPE. In this study, we mainly explored the pathogenesis of early‐onset PE.

MicroRNAs (miRNAs) are a class of small noncoding single stranded RNAs with a length of about 20–24 nucleotides in eukaryotes. They are highly conserved and stable in the evolution process. They regulate the expression of target genes by binding with 3′‐UTR of target gene mRNA, resulting in target gene degradation or inhibition of their translation, thus, playing an important role in gene expression regulation.^5^ At present, at least 500 different miRNAs are known to be expressed in placental trophoblast cells, but the biological significance of most miRNAs is still unclear.^6^ Recent studies have shown that the disorder of miRNA in placental tissues is related to the pathogenesis of PE.^7^, ^8^ Human miR‐126 gene is located in the intron 7 region of epidermal growth factor like factor 7 (EGFL‐7),^9^ while studies have found that EGFL‐7 expression sites exist in trophoblast cell lineages.^10^ Sharma et al.^11^ used in situ hybridization using a miR‐126‐specific locked nucleic acid (LNA) probe and found that miR‐126 was expressed in placental trophoblast cells and endothelial cells. Some studies have reported that miR‐126 can affect the signal transmission of vascular related factors by regulating its target gene, and then affect the invasion of trophoblast and repair of damaged blood vessels, resulting in insufficient blood supply of placental villi, resulting in reduced placental vascular bed and maternal fetal interface ischemia and hypoxia. This may be the mechanism of miR‐126 participating in PE by regulating the function of endothelial cells and trophoblasts.

VCAM‐1 is a member of the immunoglobulin superfamily, also known as CD106. It is a kind of cell adhesion molecules (CAM), which can mediate the adhesion between cells and between cells and stroma.VCAM‐1 can be expressed in the activation of blood capillary endothelial cell surface, through receptors with its matching network formation, mediating intercellular adhesion,^12^ and can also be expressed in placental villous trophoblasts, which mediates vascular infiltration of mesenchymal trophoblasts and completes vascular recasting of placental vascular bed.^13^ Biological studies have found that VCAM‐1 is the target gene of miR‐126.^14^ Some studies have indicated that miR‐126 can regulate the expression of VCAM‐1 in lung cancer, gestational diabetes mellitus, atherosclerosis, and other diseases.^15^, ^16^, ^17^ Previous studies have found that miR‐126 and VCAM‐1 are respectively involved in the occurrence and development of PE,^18^, ^19^, ^20^ however, there is no report on the correlation between miR‐126 and VCAM‐1 in PE. In this study, we detected the expression of miR‐126 and VCAM‐1 in placental tissue and trophoblast to explore the role of miR‐126 and VCAM‐1 in the pathogenesis of early‐onset PE.

## Materials and Methods

### Study population and groups

We selected 60 cases of puerpera from November 2019 to May 2020 as the research objects. This sample size was chosen based upon the results of a power analysis. All puerpera received perinatal care in the Third Affiliated Hospital of Zhengzhou University and finally underwent cesarean section. We collected the placental tissues of these women after delivery as research materials. Among them, tissues from women with EOPE were identified as the PE group (PE, *n* = 30) and those from healthy women as the normal group (N,*n* = 30). The reasons for normal parturients to choose cesarean section are abnormal fetal position, pelvic stenosis, or social factors, and so on. Then, we cultured and transfected HTR‐8/SVneo cells to investigate the functions of miR‐126 and VCAM‐1 in trophoblast cells. The study was approved by the Ethics Committee of the Third Affiliated Hospital of Zhengzhou University [(2019) Medical Lun Li No. 97]. Signed written informed consents were obtained from the patients and/or guardians. All pregnant women were excluded from multiple pregnancy, PE history or family history, hypertension, chronic hepatitis, chronic nephritis, diabetes or hereditary thrombotic tendency. The diagnostic criteria were according to the International Association for the Study of Pregnancy‐induced Hypertension (ISSHP) 2018.^21^

### Collection of placental tissues

The tissue samples were rapidly collected within 15 min after delivery of the placenta. On the maternal side, two pieces of placental villi about 1 cm^3^ in size was taken (to avoid the organization, calcification, hemorrhage lesions, and so on). One piece of the samples was quickly and repeatedly rinsed with phosphate‐buffered saline (PBS) until no blood remained, then it was snap‐frozen in liquid nitrogen, and then stored in −80°C refrigerator. The other sample was fixed with formalin and paraffin‐embedded. All operations were carried out on the ice bucket as quickly as possible, and the number of repeated freeze–thaw cycles of the samples was minimized, we also selected the 4°C to centrifuge the samples to reduce the degradation of the target RNA.

### Cell culture and transfection

The trophoblast HTR‐8/SVneo used in this experiment was purchased from the American Type Culture Collection (ATCC), and the cells were cultured in RPMI‐1640 medium (US Hyclone) containing 10% fetal bovine serum (US BI) at 37°C and 5% carbon dioxide. HTR‐8/Svneo cells were transfected with miR‐126 mimics/miR‐126 mimics NC, miR‐126 inhibitor/miR‐126 inhibitor NC (BIOFAVOR BIOTECH Co. Ltd.), respectively. For transfection, logarithmic cells were pre‐inoculated into a 6‐well plate at a dose of 2 × 10^5^ cells/well. The Lipofectamine 3000 (Invitrogen) was transfected with isoconcentration miR‐126 mimics, miR‐126 inhibitor, and NC, as per manufacturer's instructions. After incubation for 4–6 h, the supernatant was replaced with a fresh, complete medium, and the cells were kept at 37°C and 5% CO_2_. After transfection of 48 h, HTR‐8/SVneo cells were collected for the following experiments.

### Immunohistochemistry

Placental tissue was routinely dewaxed and hydrated, washed with PBS for three times, and antigen was repaired with high temperature and high pressure for 5 min. After natural cooling, the placental tissue was washed with PBS, incubated at room temperature with 50 μL of the primary antibodies against VCAM‐1 (ab134047,1:1000; Abcam) for 1 h, and incubated at room temperature with 50 μL of the secondary antibodies (1:15 000; LI‐COR) for 15 min. DAB staining, hematoxylin re‐staining, dehydration, transparency, and sealing were performed. Under the light microscope, the positive cells showed Brown granular staining in cytoplasm. The typical field of vision was selected under the microscope, and the image was processed with image pro 6.0 to calculate the optical density of the staining area.

### Reverse transcription quantitative real‐time polymerase chain reaction

From the placental tissues and cells, RNA was extracted with TRIzol reagent (Invitrogen) strictly according to the instructions. The total RNA extracted was synthesized into cDNA by reverse transcription kit (TOKOBO, Japan), and then real‐time quantitative polymerase chain reaction (qRT‐PCR) (Applied Biosystems; Thermo Fisher Scientific, Inc.) was performed with SYBR premix extaq kit (TOKOBO) and the ABI 7500 system. All experiments were carried out as quickly as possible on the ice bucket. In addition, we minimized the number of times of repeated freezing and thawing of tissues and selected a low‐temperature centrifuge to reduce the degradation of RNA. The results were expressed by 2^−△△ct^ method. The primer sequences were synthesized by tsingke Biological Technology (Zhengzhou) Co., Ltd., according to Gene Band sequences. The primers of miR‐126 and VCAM‐1 as well as the internal reference U6 and GAPDH are shown as follows:

miR‐126 (Forward: 5′‐CGTCTCGTACCGTGAGTAAT‐3′, Reverse: 5′‐GTGCAGGGTCCGAGGT‐3′)，VCAM‐1 (Forward: 5′‐CGTCTCGTACCGTGAGTAAT‐3′, Reverse: 5′‐TCAATGTGTAATTTAGCTCGGCA‐3′)，U6 (Forward: 5′‐CTCGCTTCGGCAGCACA‐3′, Reverse: 5′‐AACGCTTCACGAATTTGCGT‐3′)，GAPDH (Forward: 5′‐GGAGCGAGATCCCTCCAAAAT‐3′, Reverse: 5′‐GGCTGTTGTCATACTTCTCATGG‐3′).

### Western blot

The placental tissues and HTR‐8/SVneo cells were treated with RIPA lysis buffer (Solarbio) containing protease inhibitor to extract the protein. The extracted proteins were quantified using the BCA protein detection kit (Pierce, Appleton). Sodium dodecyl sulfate polyacrylamide gel electrophoresis (SDS‐PAGE) system was used for proteinelectrophoresis, and the protein on the gel was transferred to a PVDF membrane under low‐temperature conditions. The PVDF membrane was incubated at 4°C with the primary antibodies against VCAM‐1 (ab134047,1:1000; Abcam) and GAPDH (PAB932Hu01;1:2500 cloudclone). After that, the membrane was incubated for 1 h at RT with IRDye800CW‐labeled secondary antibodies (1:15 000; LI‐COR). Imaging was performed using a two‐color infrared laser scanning imaging system (Odyssey, LI‐COR).

### Cell invasion assay (transwell)

To conduct a cell invasion analysis, Matrigel matrix solution (BD Biosciences) diluted with 1640 was placed in 24‐pore Transwell Millipore and incubated for 37°C until gel was formed. 5 × 10^4^ HTR‐8/SVneo cells were collected, diluted with 200 μL serum‐free medium, and inoculated in the superior ventricle. The lower chamber was added with 500 μL 15% FBS medium. After incubation at 37°C for 36 h, the chamber was taken out and washed twice with PBS. The cells were fixed in methanol for 20 min and stained with crystal violet. Under the light microscope, the cells were counted in five random fields with a magnification of 200 times.

### Statistical analyses

We used statistical product and service solutions (SPSS 25.0, IBM, Armonk) statistical software for data analysis, GraphPad Prism 8.0 (Version X) and ImageJ (National Institutes of Health) were used for image editing and results analysis. Count data were presented as mean ± SE. Between‐group differences were analyzed using Student's *t*‐test or Nonparametric test. Spearman's correlation coefficient was used to analyze the association between miR‐126 expression and VCAM‐1 mRNA levels. I n all analyses, *p*<0.05 was used to indicate statistical significance.(*p < 0.05，**p < 0.01).

## Results

### Analysis of clinical data of both groups

The average age and times of pregnancy in PE group were lower than those in normal group, and BMI before pregnancy was higher than that in normal group, but the difference was not statistically significant (*p* > 0.05). However, the average gestational weeks of delivery, neonatal weight, and placental weight in PE group were lower than those in normal group (*p* < 0.01), as shown in Table 1.

**Table 1:** Clinical characteristics of normal group and preeclampsia (PE) group

Variable	N (*n* = 30)	PE (*n* = 30)	*t*	*p*
Maternal age (year)	32.10 ± 4.96	30.77 ± 5.75	0.962	>0.05
Number of pregnancies	2.97 ± 1.33	2.37 ± 1.73	1.507	>0.05
Pre‐pregnancy BMI (kg/m^2^)	23.16 ± 3.35	23.57 ± 3.09	−0.492	>0.05
Gestational age (weeks)	38.98 ± 0.49	36.72 ± 2.92	4.166	<0.01
Body weight of infant (g)	3504.33 ± 402.04	2637.83 ± 850.04	5.047	<0.01
Placental weight (g)	642.67 ± 92.47	555.73 ± 114.19	3.24	<0.01

### Expression levels of miR‐126 and VCAM‐1 mRNA in the PE and normal group

**Figure 1:**
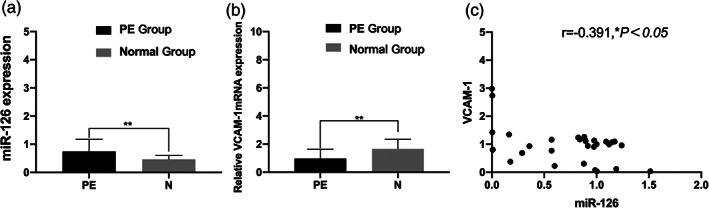
Expression of miR‐126 and vascular endothelial cell adhesion molecule‐1 (VCAM‐1) mRNA in the preeclampsia (PE) and normal group. Results of qRT‐PCR reveal that the expression level of miR‐126 in the PE group is higher than that in the normal group (a,**p < 0.01), the relative expression level of VCAM‐1 mRNA in the PE group is lower than that in the normal group (b,**p<0.01), both the difference were statistically significant; correlation between miR‐126 and VCAM‐1 mRNA expression levels in placental tissues in patients with PE (c, *r* = −0.391,*p < 0.05).

The expression level of miR‐126 in the PE group was significantly higher than that in the normal group (Figure 1a). However, the expression level of VCAM‐1 mRNA was significantly lower in placental tissues from PE group compared with the normal group(Figure 1b). Both the difference were statistically significant (*p* < 0.01). According to Spearman's correlation analysis, there was a negative correlation between the relative expression levels of VCAM‐1 mRNA and miR‐126 in the PE group (*r* = −0.391, *p* < 0.05;Figure 1c).

### Expression levels of VCAM‐1 protein in placental tissues

**Figure 2:**
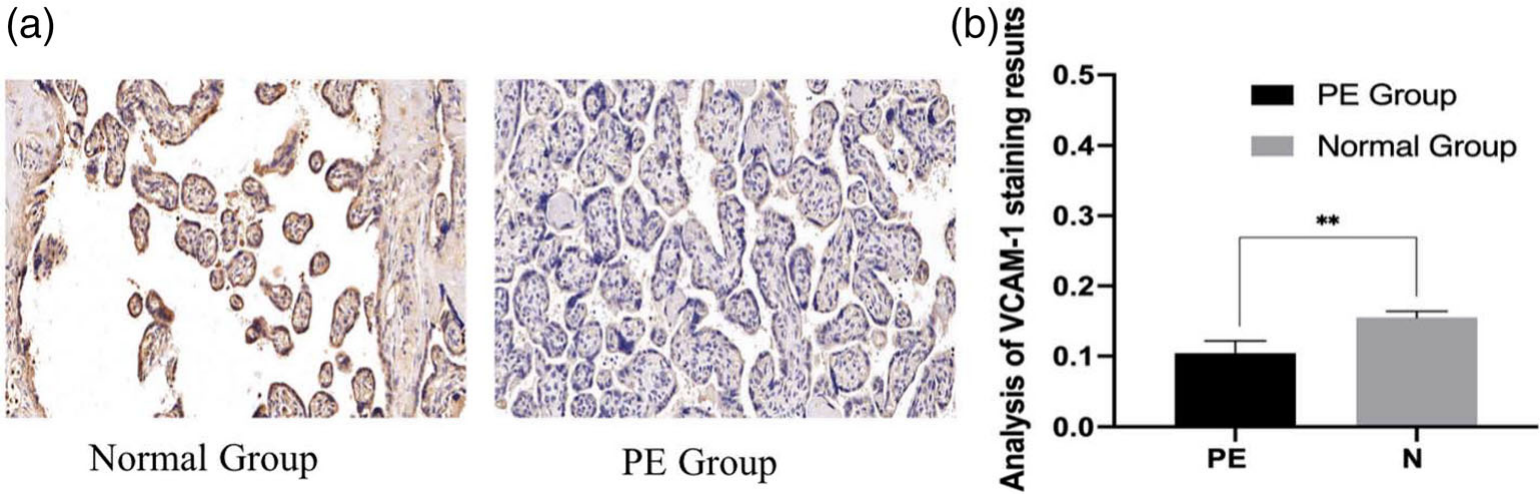
Location analysis of vascular endothelial cell adhesion molecule‐1 (VCAM‐1) in placental tissues of two groups，VCAM‐1 was expressed in both groups of placental tissues and was mainly localized in the cell membrane and cytoplasm of trophoblast cells (a). Analysis of optical density values by ImageJ software showed that the expression of VCAM‐1 was different in the two groups, and was lower in the preeclampsia (PE) group than in the Normal group (b,**p < 0.01)

Immunohistochemical results showed that VCAM‐1 was expressed in both normal group and PE group, and it was mainly expressed in trophoblast cell membrane and cytoplasm (Figure 2a). ImageJ software image analysis results showed that compared with the normal group, VCAM‐1 expression was significantly decreased in the PE group (Figure 2b, *p* < 0.01).

**Figure 3:**
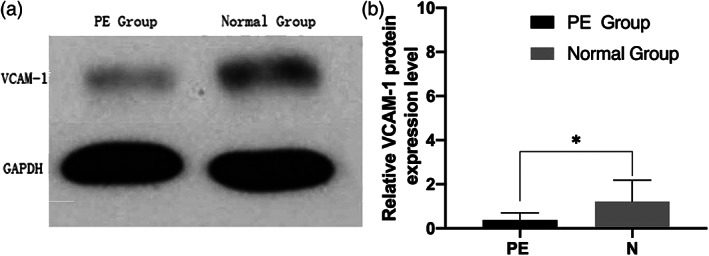
Expression levels of vascular endothelial cell adhesion molecule‐1 (VCAM‐1) protein in placental tissues, representative Western blot of VCAM‐1 protein levels (a), the grayscale scanning analysis of ImageJ software showed that the expression levels of the two groups were significantly different (b,*p < 0.05)

Western blot results showed that the expression of VCAM‐1 protein in placental tissues of pregnant women in PE group was significantly lower than that in normal group (Figure 3a). Gray scanning analysis showed that the relative gray value of VCAM‐1 protein expression in placental tissues of the PE group was lower than that of the normal group (Figure 3b, *p* < 0.05).

### Transfection miR‐126

**Figure 4:**
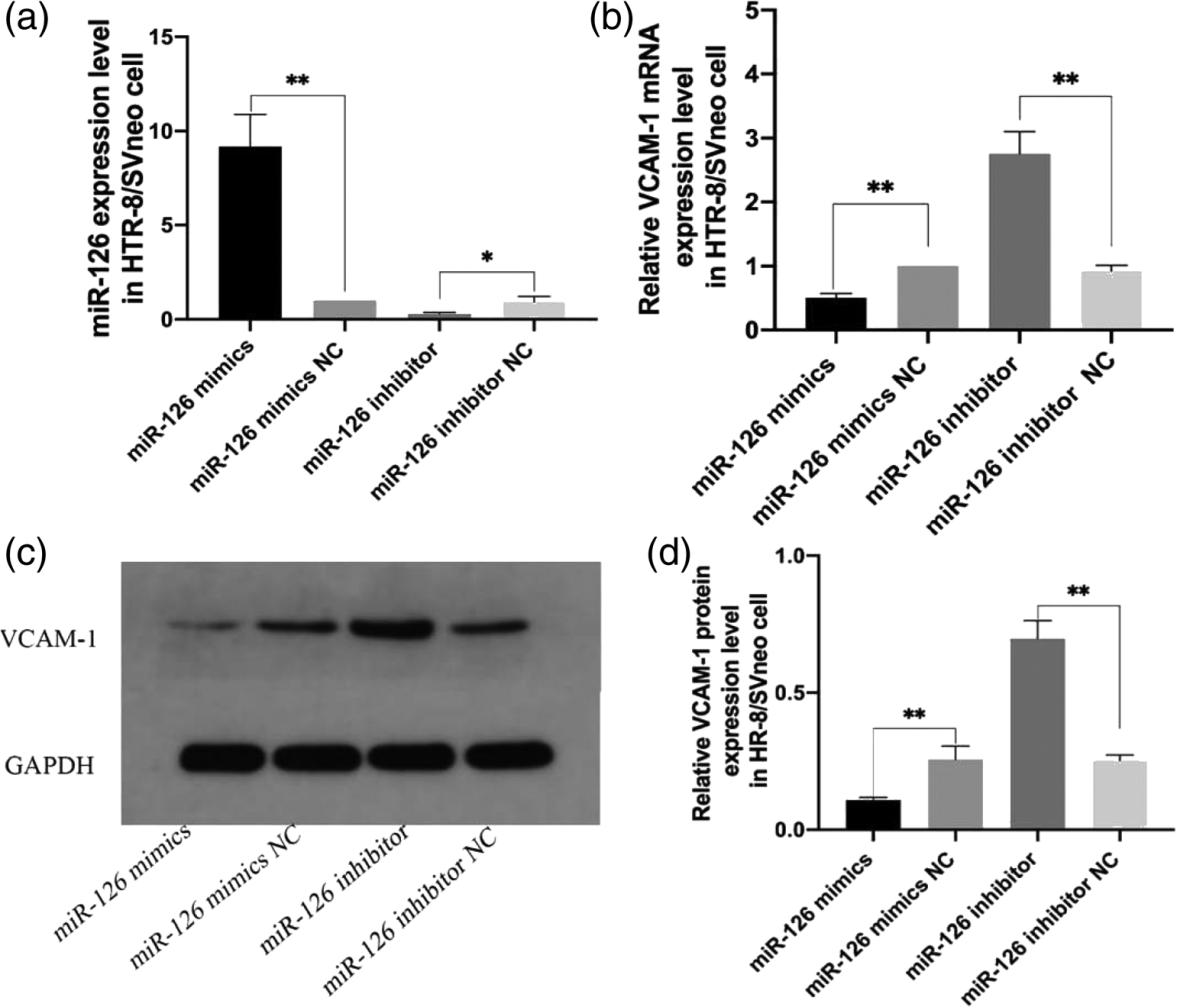
The expression of miR‐126 and vascular endothelial cell adhesion molecule‐1 (VCAM‐1) after transfection of miR‐126 mimics or miR‐126 inhibitor. PCR results showed the changes of VCAM‐1 mRNA expression in HTR‐8/SVneo cells after transfection with miR‐126 (a,b,**p < 0.01); comparison of VCAM‐1 protein expression levels in HTR‐8/SVneo cell after transfection with miR‐126 (c,d,**p < 0.01).The NC is negative control, miR‐126 mimics NC and miR‐126 inhibitor NC is a negative control group containing a non‐specific interference fragment.

Compared with the miR‐126 mimics NC group, after transfection with miR‐126 mimics, the expression level of VCAM‐1 mRNA was significantly decreased (Figure 4a,b, *p* < 0.01). We used Image J software to visualize the gray value of WB band, obtained the relative expression of VCAM‐1 in each group of cells after transfection, and then analyzed the difference between the groups with nonparametric test. Compared with the miR‐126 inhibitor NC group, the VCAM‐1 protein expression level was significantly increased in miR‐126 inhibitor group, as shown in (Figure 4c,d *p* < 0.01).

### Effects of transfection miR‐126 on invasion of HTR8/SVneo cells

**Figure 5:**
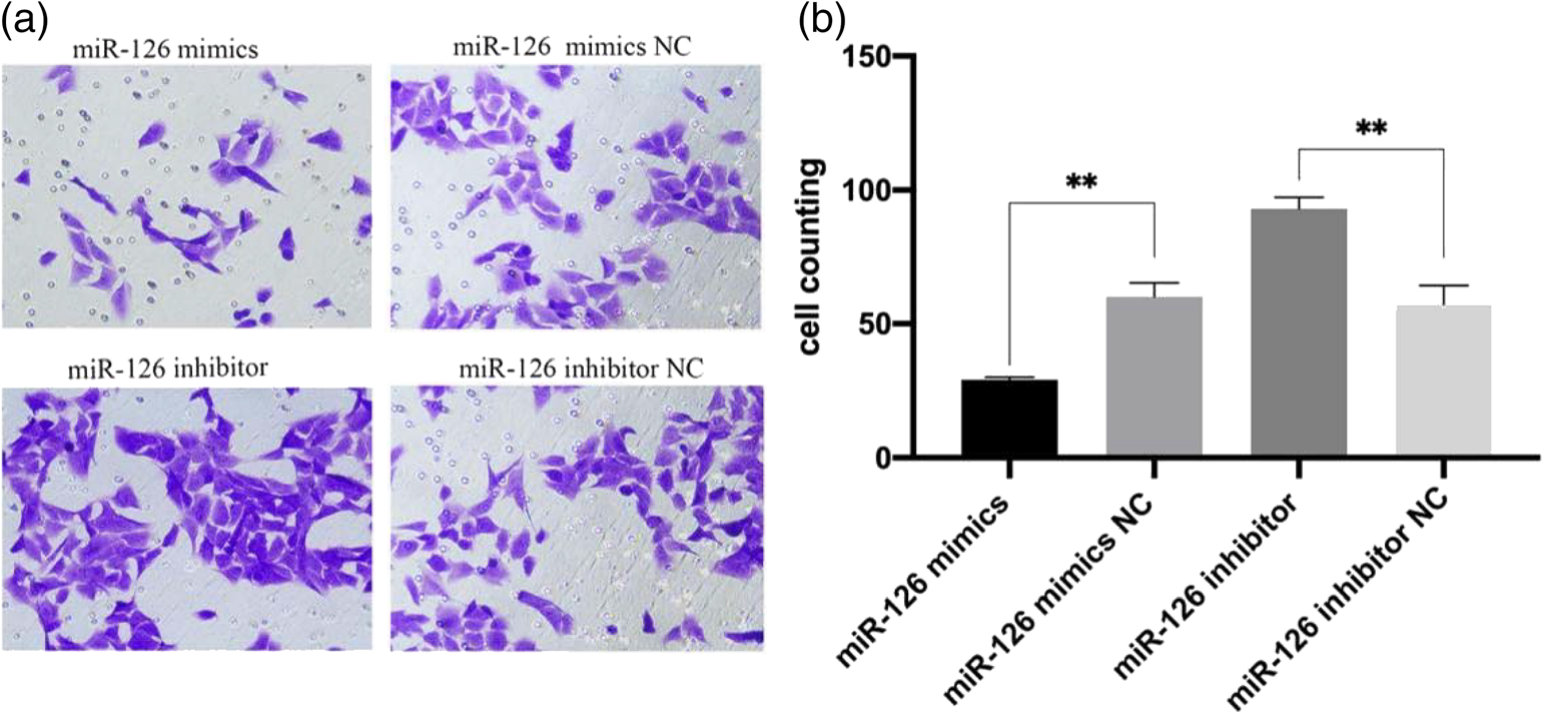
Transwell assay results showed the invasion effect of HTR‐8/SVneo cells after transfection with miR‐126 (a, magnification: 200). Compared with the NC group, the invasion ability of transfected miR‐126 mimics cells was reduced, while that of transfected miR‐126 inhibitor cells was increased (b,**p < 0.01).

The results of Transwell invasion assay showed that compared with the NC group, the number of invasive cells of HTR‐8/SVneo cells was significantly decreased after transfection of miR‐126 mimics，the opposite was true for transfection with miR‐126 inhibitor (Figure 5, *p* < 0.05).

## Discussion

EOPE is called “placental origin” disease, the pathogenesis mainly consists of inadequate extravilloustrophoblasts (EVTs) infiltration, obstruction of maternal placenta construction, and vascular recast, which leads to the ischemia and hypoxia of the mother‐fetus interface, triggering a series of oxidative stress and endoplasmic areolar stress, and finally affecting the fetal blood supply.^22^ EOPE has a negative impact on perinatal maternal and child health as well as long‐term maternal and child disease,^23^, ^24^so further study of its pathogenesis is worthwhile.

In recent years, studies on early‐onset PE have gradually deepened into the molecular mechanism, and the role of miRNA in PE cannot be ignored.^18^, ^25^ However, the pathway of action of miRNA is still unclear, so we studied the role of miR‐126 and VCAM‐1 in EOPE placental tissues and trophoblast cells, providing the possibility to elucidate the pathogenesis and treatment of EOPE.

In this study, compared with the normal control group, the expression level ofmiR‐126 in maternal placental tissues of the PE group was higher, and the difference was statistically significant. It indicated that miR‐126 was involved in the onset of PE, which was consistent with the microRNA sequencing results of placental tissues of PE patients by Yang et al.^19^ It was speculated that the high expression of miR‐126 could accelerate the apoptosis of endothelial cells, resulting in the destruction of vascular endothelial integrity, structural, and functional damage, and the increase of vasoconstrictor factors, vascular pressure, and blood pressure, and finally the development of PE.^26^ The results of this experiment showed that VCAM‐1 was mainly distributed in the cytoplasm and membrane of trophoblast cells, and the expression level of VCAM‐1 in EOPE placental tissues was lower than that in the normal group, with significant difference (*p* < 0.01). Correlation analysis showed that miR‐126 was negatively correlated with VCAM‐1 in the placental tissues of patients with PE (*R* = −0.391, *p* < 0.05). As an adhesion molecule, VCAM‐1 plays a very important role in the adhesion process of trophoblast cells, and the change of biological behavior of trophoblast cells may be related to the abnormal expression of related genes, leading to proteins or molecules related to trophoblast cell invasion. Biological information shows that VCAM‐1 is a target protein of miR‐126.^14^ Kwee et al.^27^ found that mice with VCAM‐1 deletion had severe defects in placental development, leading to death. An appropriate amount of VCAM‐1 can maintain the stable state of blood vessels and induce and strengthen the infiltration of trophoblast cells into the decidual layer, muscle layer, and blood vessels, which not only makes the placenta adhere firmly, but also provides favorable conditions for the exchange of oxygen and nutrients between mother and fetus. Therefore, we speculate that the expression of miR‐126 in EOPE is negatively correlated with the expression of VCAM‐1, which may reduce the adhesion and transformation between trophoblast cells and vascular endothelial cells, myoma uteri, and so on, weaken the invasion ability of trophoblast cells, make the placental vessels recast deficient and placental shallow implantation, and finally lead to the occurrence of PE.

Normal invasion of trophoblast cells during pregnancy is a key step to ensure adequate blood flow and nutrient exchange between mother and fetus. Abnormal invasion of trophoblast cells will lead to pathological placenta.^28^ To further confirm the effects of miR‐126 and VCAM‐1 on trophoblast cells, we conducted in vitro functional experiments. The results confirmed that compared with the NC group, VCAM‐1 expression level was decreased in HTR‐8/SVneo cells transfected with miR‐126 mimics, and increased in the miR‐126 inhibitor group as shown in Figure 4. Then, we tested the invasion function of the transfected cells, and the results showed that miR‐126 overexpression could significantly inhibit the invasion ability of HTR‐8/SVneo cells as shown in Figure 5. Therefore, we hypothesized that miR‐126 may be involved in the occurrence and development of EOPE by regulating the expression of VCAM‐1, thereby affecting the invasion function of trophoblast cells.

The most effective treatment for EOPE is timely termination of pregnancy,^3^ but for EOPE, early termination of pregnancy increases the risk of neonatal disease, so finding an effective screening and treatment method is a key and difficult issue for obstetricians. Some studies have found that targeted knockout of some pathogenic mirnas in patients can better improve patient outcomes.^29^ Therefore, miR‐126 targeted therapy for patients with PE may become a reality, which will be the content of our follow‐up studies.

In summary, On the one hand, miR‐126 overexpression in EOPE may cause vascular endothelial injury and damage placental neovascularization by promoting endothelial cell apoptosis. On the other hand, VCAM‐1 expression may be regulated to inhibit the invasion function of trophoblast cells, resulting in uterine vascular recasting disorder, shallow placental implantation, and participating in the occurrence and development of PE. This study provides a certain reference value for the comprehensive understanding of the role and relationship between miR‐126 and VCAM‐1，and provide a new direction for targeted therapy of PE.

## Disclosure

There are no financial conflicts of interest to disclose.

## References

[1] Duley L . The global impact of pre‐eclampsia and eclampsia. Semin Perinatol. 2009;33(3):130–7. 19464502 10.1053/j.semperi.2009.02.010

[2] Tavana Z , Hosseinmirzaei S . Comparison of maternal serum magnesium level in pre‐eclampsia and Normal pregnant women. Iran Red Crescent Med J. 2013;15(12):e10394. 24693379 10.5812/ircmj.10394 PMC3955494

[3] Amaral LM , Wallace K , Owens M , LaMarca B . Pathophysiology and current clinical management of preeclampsia. Curr Hypertens Rep. 2017;19(8):61. 28689331 10.1007/s11906-017-0757-7 PMC5916784

[4] Madazli R , Yuksel MA , Imamoglu M , Tuten A , Oncul M , Aydin B , et al. Comparison of clinical and perinatal outcomes in early‐ and late‐onset preeclampsia. Arch Gynecol Obstet. 2014;290(1):53–7. 24549271 10.1007/s00404-014-3176-x

[5] Carthew RW , Sontheimer EJ . Origins and mechanisms of miRNAs and siRNAs. Cell. 2009;136(4):642–55. 19239886 10.1016/j.cell.2009.01.035 PMC2675692

[6] Mouillet JF , Ouyang Y , Coyne CB , Sadovsky Y . MicroRNAs in placental health and disease. Am J Obstet Gynecol. 2015;213(4 Suppl):S163–72. 26428496 10.1016/j.ajog.2015.05.057 PMC4592520

[7] Choi SY , Yun J , Lee OJ , Han HS , Yeo MK , Lee MA , et al. MicroRNA expression profiles in placenta with severe preeclampsia using a PNA‐based microarray. Placenta. 2013;34(9):799–804. 23830491 10.1016/j.placenta.2013.06.006

[8] Betoni JS , Derr K , Pahl MC , Rogers L , Muller CL , Packard RE , et al. MicroRNA analysis in placentas from patients with preeclampsia: comparison of new and published results. Hypertens Pregnancy. 2013;32(4):321–39. 23844600 10.3109/10641955.2013.807819

[9] Nikolic I , Plate KH , Schmidt MHH . EGFL7 meets miRNA‐126: an angiogenesis alliance. J Angiogenes Res. 2010;2(1):9. 20529320 10.1186/2040-2384-2-9 PMC2901201

[10] Lacko LA , Massimiani M , Sones JL , Hurtado R , Salvi S , Ferrazzani S , et al. Novel expression of EGFL7 in placental trophoblast and endothelial cells and its implication in preeclampsia. Mech Dev. 2014;133:163–76. 24751645 10.1016/j.mod.2014.04.001 PMC4177412

[11] Sharma A , Lacko LA , Argueta LB , Glendinning MD , Stuhlmann H . miR‐126 regulates glycogen trophoblast proliferation and DNA methylation in the murine placenta. Dev Biol. 2019;449(1):21–34. 30771304 10.1016/j.ydbio.2019.01.019 PMC6451886

[12] Dietmaier W , Hartmann A , Wallinger S , Heinmöller E , Kerner T , Endl E , et al. Multiple mutation analyses in single tumor cells with improved whole genome amplification. Am J Pathol. 1999;154(1):83–95. 9916922 10.1016/S0002-9440(10)65254-6 PMC1853424

[13] Zhou Y , Damsky CH , Fisher SJ . Preeclampsia is associated with failure of human cytotrophoblasts to mimic a vascular adhesion phenotype. One cause of defective endovascular invasion in this syndrome? J Clin Invest. 1997;99(9):2152–64. 9151787 10.1172/JCI119388 PMC508045

[14] Harris TA , Yamakuchi M , Ferlito M , Mendell JT , Lowenstein CJ . MicroRNA‐126 regulates endothelial expression of vascular cell adhesion molecule 1. Proc Natl Acad Sci U S A. 2008;105(5):1516–21. 18227515 10.1073/pnas.0707493105 PMC2234176

[15] Fu X , Niu T , Li X . MicroRNA‐126‐3p attenuates Intracerebral hemorrhage‐induced blood‐brain barrier disruption by regulating VCAM‐1 expression. Front Neurosci. 2019;13:866. 31474826 10.3389/fnins.2019.00866 PMC6707088

[16] Chen M , Peng W , Hu S , Deng J . miR‐126/VCAM‐1 regulation by naringin suppresses cell growth of human non‐small cell lung cancer. Oncol Lett. 2018;16(4):4754–60. 30197681 10.3892/ol.2018.9204 PMC6126337

[17] Pan X , Hou R , Ma A , Wang T , Wu M , Zhu X , et al. Atorvastatin Upregulates the expression of miR‐126 in apolipoprotein E‐knockout mice with carotid atherosclerotic plaque. Cell Mol Neurobiol. 2017;37(1):29–36. 26886754 10.1007/s10571-016-0331-x PMC11482060

[18] Frazier S , McBride MW , Mulvana H , Graham D . From animal models to patients: the role of placental microRNAs, miR‐210, miR‐126, and miR‐148a/152 in preeclampsia. Clin Sci (Lond). 2020;134(8):1001–25. 32337535 10.1042/CS20200023 PMC7239341

[19] Yang S , Li H , Ge Q , Guo L , Chen F . Deregulated microRNA species in the plasma and placenta of patients with preeclampsia. Mol Med Rep. 2015;12(1):527–34. 25738738 10.3892/mmr.2015.3414

[20] Wang Y , Zhang X , Cheng GM , Ren CC . Expression of transforming growth factor‐beta 1, vascular cell adhesion molecule‐1 and endothelium‐selectin in placenta of patients with pre‐eclampsia. Zhonghua Fu Chan Ke Za Zhi. 2006;41(8):514–7. 17083832

[21] Brown MA , Magee LA , Kenny LC , Karumanchi SA , McCarthy F , Saito S , et al. The hypertensive disorders of pregnancy: ISSHP classification, diagnosis & management recommendations for international practice. Pregnancy Hypertens. 2018;13:291–310. 29803330 10.1016/j.preghy.2018.05.004

[22] Steegers EA , von Dadelszen P , Duvekot JJ , Pijnenborg R . Pre‐eclampsia. Lancet. 2010;376(9741):631–44. 20598363 10.1016/S0140-6736(10)60279-6

[23] Enkhmaa D , Wall D , Mehta PK , Stuart JJ , Rich‐Edwards JW , Merz CNB , et al. Preeclampsia and vascular function: a window to future cardiovascular disease risk. J Womens Health (Larchmt). 2016;25(3):284–91. 26779584 10.1089/jwh.2015.5414 PMC4790201

[24] Lu HQ , Hu R . Lasting effects of intrauterine exposure to preeclampsia on offspring and the underlying mechanism. AJP Rep. 2019;9(3):e275–91. 31511798 10.1055/s-0039-1695004 PMC6736667

[25] Biró O , Rigó J Jr . The pathogenetic role and expression profile of microRNAs in preeclampsia. Orv Hetil. 2018;159(14):547–56. 29611751 10.1556/650.2018.31025

[26] Possomato‐Vieira JS , Khalil RA . Mechanisms of endothelial dysfunction in hypertensive pregnancy and preeclampsia. Adv Pharmacol. 2016;77:361–431. 27451103 10.1016/bs.apha.2016.04.008 PMC4965238

[27] Kwee L , Baldwin HS , Shen HM , Stewart CL , Buck C , Buck CA , et al. Defective development of the embryonic and extraembryonic circulatory systems in vascular cell adhesion molecule (VCAM‐1) deficient mice. Development. 1995;121(2):489–503. 7539357 10.1242/dev.121.2.489

[28] Knöfler M , Pollheimer J . IFPA award in Placentology lecture: molecular regulation of human trophoblast invasion. Placenta. 2012;33 Suppl(2):S55–62. 22019198 10.1016/j.placenta.2011.09.019 PMC3272142

[29] Rupaimoole R , Slack FJ . MicroRNA therapeutics: towards a new era for the management of cancer and other diseases. Nat Rev Drug Discov. 2017;16(3):203–22. 28209991 10.1038/nrd.2016.246

